# Applying artificial intelligence and digital health technologies, Viet Nam

**DOI:** 10.2471/BLT.22.289423

**Published:** 2023-05-26

**Authors:** Ho Quang Chanh, Damien K Ming, Quang Huy Nguyen, Tran Minh Duc, Luu Phuoc An, Huynh Trung Trieu, Stefan Karolcik, Bernard Hernandez, Perez, Jennifer Van Nuil, Ngan Nguyen Lyle, Evelyne Kestelyn, Louise Thwaites, Pantelis Georgiou, Chris Paton, Alison Holmes, Nguyen Van Vinh Chau, Sophie Yacoub

**Affiliations:** a Oxford University Clinical Research Unit, 764 Vo Van Kiet Street, District 5, Ho Chi Minh City, 72708, Viet Nam.; b Centre for Antimicrobial Optimisation, Imperial College London, England.; c Hospital for Tropical Diseases, Ho Chi Minh City, Viet Nam.; d Centre for Bio Inspired Technology, Imperial College London, England.; e Department of Information Science, University of Otago, New Zealand.; f Department of Health of Ho Chi Minh City, Viet Nam.

## Abstract

**Problem:**

Direct application of digital health technologies from high-income settings to low- and middle-income countries may be inappropriate due to challenges around data availability, implementation and regulation. Hence different approaches are needed.

**Approach:**

Within the Viet Nam ICU Translational Applications Laboratory project, since 2018 we have been developing a wearable device for individual patient monitoring and a clinical assessment tool to improve dengue disease management. Working closely with local staff at the Hospital for Tropical Diseases, Ho Chi Minh City, we developed and tested a prototype of the wearable device. We obtained perspectives on design and use of the sensor from patients. To develop the assessment tool, we used existing research data sets, mapped workflows and clinical priorities, interviewed stakeholders and held workshops with hospital staff.

**Local setting:**

In Viet Nam, a lower middle-income country, the health-care system is in the nascent stage of implementing digital health technologies.

**Relevant changes:**

Based on patient feedback, we are altering the design of the wearable sensor to increase comfort. We built the user interface of the assessment tool based on the core functionalities selected by workshop attendees. The interface was subsequently tested for usability in an iterative manner by the clinical staff members.

**Lessons learnt:**

The development and implementation of digital health technologies need an interoperable and appropriate plan for data management including collection, sharing and integration. Engagements and implementation studies should be conceptualized and conducted alongside the digital health technology development. The priorities of end-users, and understanding context and regulatory landscape are crucial for success.

## Résumé

**Problème:**

Transposer directement des technologies numériques en matière de santé des pays à revenu élevé vers des pays à revenu faible et intermédiaire peut se révéler inapproprié en raison des défis que posent la disponibilité, la mise en œuvre et la réglementation des données. Par conséquent, différentes approches sont nécessaires.

**Approche:**

Dans le cadre du projet Viet Nam ICU Translational Applications Laboratory, nous développons depuis 2018 un dispositif portable pour le suivi individuel des patients, ainsi qu'un outil d'évaluation clinique visant à améliorer la gestion de la dengue. En étroite collaboration avec les équipes locales de l'Hôpital des maladies tropicales, à Ho Chi Minh Ville, nous avons mis au point et testé un prototype de ce dispositif portable. Nous avons ainsi pu recueillir l'avis des patients quant à la conception et à l'utilisation du capteur. Pour élaborer l'outil d'évaluation, nous avons employé des ensembles de données de recherche existantes, cartographié les processus et les priorités cliniques, interrogé les parties prenantes et organisé des ateliers avec le personnel de l'hôpital.

**Environnement local:**

Le Viet Nam est un pays à revenu faible, où le déploiement des technologies numériques n'en est encore qu'à ses débuts dans le secteur des soins de santé.

**Changements significatifs:**

En nous fondant sur les avis des patients, nous avons modifié le design du capteur portable en vue de le rendre plus confortable. Nous avons également imaginé l'interface utilisateur de l'outil d'évaluation en nous inspirant des principales fonctionnalités sélectionnées par les participants aux ateliers. Cette interface a ensuite été testée de manière itérative par les membres du personnel clinique afin d'en mesurer la facilité d'utilisation.

**Leçons tirées:**

L'élaboration et la mise en œuvre des technologies numériques en matière de santé requièrent un plan de gestion des données adéquat et interopérable, notamment en termes de collecte, de partage et d'intégration. Des études sur l'engagement et le déploiement doivent être conceptualisées et menées parallèlement au développement de telles technologies. Tenir compte des priorités des utilisateurs finaux, mais aussi comprendre le contexte et le cadre réglementaire est essentiel à la réussite de ce projet.

## Resumen

**Situación:**

La aplicación directa de las tecnologías sanitarias digitales desde los entornos de ingresos altos a los países de ingresos bajos y medios puede resultar inadecuada debido a los problemas relacionados con la disponibilidad de datos, la implementación y la regulación. Por consiguiente, se necesitan enfoques diferentes.

**Enfoque:**

Dentro del proyecto Viet Nam ICU Translational Applications Laboratory, se está desarrollando desde 2018 un dispositivo ponible para la monitorización individual del paciente y una herramienta de evaluación clínica para mejorar el manejo de la enfermedad del dengue. En estrecha colaboración con el personal local del Hospital para las Enfermedades Tropicales de Ciudad Ho Chi Minh, se desarrolló y probó un prototipo del dispositivo ponible. Se obtuvo la opinión de los pacientes sobre el diseño y el uso del sensor. Para desarrollar la herramienta de evaluación, se utilizaron conjuntos de datos de investigación existentes, se trazaron flujos de trabajo y prioridades clínicas, se entrevistó a las partes interesadas y se organizaron talleres con el personal del hospital.

**Marco regional:**

En Vietnam, un país de ingresos medios bajos, el sistema sanitario se encuentra en una fase incipiente de implantación de tecnologías sanitarias digitales.

**Cambios importantes:**

A partir de los comentarios de los pacientes, se ha modificado el diseño del sensor portátil para aumentar su comodidad. Se creó la interfaz de usuario de la herramienta de evaluación según las principales funciones que seleccionaron los asistentes al taller. Posteriormente, los miembros del personal clínico comprobaron la usabilidad de la interfaz de manera iterativa.

**Lecciones aprendidas:**

El desarrollo y la implementación de la tecnología sanitaria digital necesitan un plan interoperable y adecuado para la gestión de datos, que incluya la recopilación, el intercambio y la integración. Los compromisos y los estudios de implementación deben conceptualizarse y llevarse a cabo junto con el desarrollo de la tecnología sanitaria digital. Las prioridades de los usuarios finales y la comprensión del contexto y el panorama reglamentario son fundamentales para lograr los objetivos.

## ملخص

**المشكلة:**

إن التطبيق المباشر لتقنيات الصحة الرقمية من الأوضاع بالدول ذات الدخل المرتفع إلى الدول ذات الدخل المنخفض إلى المتوسط، قد يكون غير مناسب بسبب التحديات المتعلقة بتوافر البيانات، وتنفيذها، وتنظيمها. ومن ثم فإن هناك حاجة إلى أساليب مختلفة.

**الأسلوب:**

نحن نقوم ضمن مشروع مختبر التطبيقات الانتقالية لوحدة العناية المركزة في فييت نام، منذ عام 2018 بتطوير جهاز يمكن ارتداؤه لمراقبة المرضى من الأفراد، وأداة للتقييم الإكلينيكي لتحسين إدارة مرض حمى الضنك. من خلال العمل عن كثب مع الموظفين المحليين في مستشفى الأمراض الاستوائية، هو تشي من سيتي، قمنا بتطوير نموذج أولي للجهاز القابل للارتداء واختباره. حصلنا على منظور لتصميم واستخدام المستشعر من المرضى. لتطوير أداة التقييم، استخدمنا مجموعات بيانات البحث الحالية، وخططنا عمليات سير العمل والأولويات الإكلينيكية، وأجرينا مقابلات مع أصحاب المصلحة، وعقدنا ورش عمل مع موظفي المستشفى.

**المواقع:**

المحلية فييت نام هي دولة ذات دخل منخفض إلى متوسط، ويعد نظام الرعاية الصحية فيها المرحلة الوليدة لتنفيذ تقنيات الصحة الرقمية.

**التغيّرات ذات الصلة:**

بناءً على ملاحظات المريض، نقوم بتغيير تصميم المستشعر القابل للارتداء لزيادة الراحة. قمنا بوضع واجهة المستخدم الخاصة بأداة التقييم بناءً على الوظائف الأساسية المختارة للحاضرين في ورشة العمل. تم اختبار الواجهة لاحقًا للتحقق من قابليتها للاستخدام بطريقة تكرارية بواسطة أعضاء الفريق الإكلينيكي.

**الدروس المستفادة:**

إن تطوير تقنية الصحة الرقمية وتنفيذها يحتاج إلى خطة مناسبة وقابلة للتشغيل المتبادل لإدارة البيانات بما في ذلك التجمع والمشاركة والتكامل. يجب وضع تصور لعمليات المشاركة ودراسات التنفيذ، وإجرائها جنبًا إلى جنب مع تطوير تقنية الصحة الرقمية. تعتبر أولويات المستخدمين النهائيين، وفهم السياق والمشهد التنظيمي، أمرًا بالغ الأهمية لتحقيق النجاح.

## 摘要

**问题:**

由于数据可用性、实施和监管方面存在的挑战，将数字健康技术从高收入环境直接应用到中低收入国家可能并不合适。因此，需要采用不同的方法。

**方法:**

在越南 ICU 转化应用实验室项目中，我们自 2018 年以来一直在开发用于个体患者监测的可穿戴设备和临床评估工具，以改善登革热疾病管理。我们与胡志明市热带病医院的当地工作人员开展密切合作，开发并测试了可穿戴设备原型。我们获得了患者对传感器设计和使用的看法。为了开发评估工具，我们采用了现有的研究数据集，制定了工作流程和临床优先事项，采访了利益相关者，并与医院工作人员一起举行了研讨会。

**当地状况:**

在中低收入国家越南，卫生保健系统正处于实施数字健康技术的初级阶段。

**相关变化:**

根据患者反馈，我们正在改进可穿戴传感器的设计以提升舒适度。我们根据研讨会与会者选择的核心功能构建了评估工具的用户界面。随后，临床工作人员通过迭代方法测试了该界面的可用性。

**经验教训:**

数字健康技术的开发和实施需要一个可互操作且适当的数据管理计划，包括数据的收集、共享和集成。应将参与和实施研究概念化，并与数字健康技术的开发一起进行。终端用户的优先事项以及对背景和监管环境的了解对成功至关重要。

## Резюме

**Проблема:**

Прямое применение технологий цифрового здравоохранения, заимствованных из опыта стран с высоким уровнем дохода, может быть нецелесообразным в странах с низким и средним уровнем дохода из-за сложностей, связанных с доступностью данных, внедрением и регулированием. Поэтому необходимы различные подходы.

**Подход:**

В рамках проекта лаборатории трансляционных приложений для ОИТ во Вьетнаме с 2018 года разрабатывается носимое устройство для индивидуального мониторинга пациентов и инструмент клинической оценки для более эффективного ведения болезни денге. В тесном сотрудничестве с местным персоналом больницы тропических болезней в г. Хошимине был разработан и протестирован прототип носимого устройства. От пациентов были получены сведения о конструкции и использовании датчика. Для разработки инструмента оценки были использованы доступные наборы данных исследований, сопоставлены рабочие процессы и клинические приоритеты, опрошены заинтересованные стороны и проведены семинары с участием персонала больницы.

**Местные условия:**

Во Вьетнаме, который относится к странам со средненизким уровнем дохода, система здравоохранения находится на начальной стадии внедрения технологий цифрового здравоохранения.

**Осуществленные перемены:**

На основании отзывов пациентов с целью повышения удобства использования была изменена конструкция носимого датчика. На основании выбранных участниками семинара основных функциональных возможностей был разработан пользовательский интерфейс инструмента оценки. Впоследствии интерфейс был протестирован на удобство использования итеративным способом сотрудниками клиники.

**Выводы:**

Для разработки и внедрения технологий цифрового здравоохранения необходим функционально совместимый и соответствующий план управления данными, включая сбор, обмен и интеграцию. Исследования по вовлечению и внедрению должны разрабатываться и проводиться параллельно с разработкой технологий цифрового здравоохранения. Приоритеты конечных пользователей и понимание контекста и нормативно-правовой базы имеют решающее значение для достижения успеха.

## Introduction

Artificial intelligence and digital health technologies are improving clinical services, and are facilitating the decentralization of health care. Within low- and middle-income countries, opportunities for applying these innovations are rapidly being realized for diagnostics, patient monitoring and aiding clinical decision-making.^1^ However, significant barriers to implementation remain, including data sharing and individual rights to privacy, algorithmic bias, end-user interpretability and accountability.^2^ To date, research addressing these barriers has mainly been conducted in high-income settings,^3^ and direct application of these findings to low-and middle-income health-care settings is often inappropriate, given significant differences in context and resource availability.^2^^,^^4^

Here, we share our experience in conducting multidisciplinary research for the development and implementation of digital health technologies in Viet Nam. We outline challenges encountered and describe specific findings around real-world implementation. Our approaches and methods are applicable to similar settings and provide insights on design considerations from a low- and middle-income country perspective. 

## Local setting 

In Viet Nam, a lower middle-income country, opportunities exist to apply artificial intelligence and digital health technologies to improve health outcomes, especially for many infectious diseases, which exert significant burdens on the health-care system. The Vietnamese health-care system is in the nascent stage of implementing digital health technologies.^5^ For example, electronic medical records are being introduced for routine clinical use. In contrast with high-income settings where electronic medical records data is routinely generated, availability of such data in our setting is limited. While information on heterogeneity between health-care settings exists, care pathways, strategic health system priorities, infrastructure and capacity for individual settings remain poorly characterized, hampering the estimation of resources required and the development of an implementation plan of new digital health technologies.^2^^,^^4^

## Approach

The Viet Nam ICU Translational Applications Laboratory (VITAL) project, funded by the Wellcome Trust, started in 2018. The project aims to reduce morbidity and mortality from infectious diseases by using innovative technology and clinical approaches to improve the management of critical care. This multidisciplinary project is a collaboration between the Hospital for Tropical Diseases; Oxford University Clinical Research Unit in Ho Chi Minh City; Imperial College London; University of Oxford and other partners. The implementation site is the 550-bed public Hospital for Tropical Diseases, which is a referral centre for infectious diseases for the south of Viet Nam.

As a part of the project, we have been conducting research and implementation studies to investigate the role and utility of novel digital health technologies for improving the management of dengue, a major public health issue in Viet Nam. Our work includes the development of a novel wearable, and an artificial intelligence-driven electronic clinical decision support system, namely D-SCAPE (dengue severity classification and prediction wearable) and D-CAT (dengue clinical assessment tool), respectively.

In collaboration with engineers at Imperial College London, we developed a wearable prototype that could optically obtain plethysmograms. While this first protoype did not display any data collected, our aim is that future protypes will show pulse, oxygen saturation and trends in haematocrit levels. We tested the prototype on 50 dengue patients admitted to the implementation hospital, and we explored patients’ perspectives on design and use of the sensor. We also collected physiological data including pulse, pulse wave and oxygen saturation, from 250 hospitalized dengue patients with different severity grades, using a commercial wearable named SmartCare (SmartCare Analytics, Oxford, England) to develop artificial intelligence models for dengue physiological monitoring.

The development of the assessment tool consisted of training and testing artificial intelligence models alongside implementation work, which included landscape exploration, user interface design and stakeholder usability testing. To develop the artificial intelligence predictive algorithms, we used existing research data sets, including pooled data from 8000 Vietnamese patients collected over 19 years.^6^ Concurrently, we conducted a multistage qualitative study to better understand existing health-care pathways and the possible impact of the interventions on these pathways. First, we mapped workflow and clinical priorities in our setting to characterize differences in management between patient cohorts which could affect deployment.^7^ We then organized 10 in-person interviews and two workshops with hospital staff who directly care for dengue patients, to explore their perspectives towards dengue and their needs and desired functionalities of tools for improving disease management. We used the information obtained to guide the system frontend design. Finally, we conducted 50 separate observations of 15 clinicians to evaluate the usability of the assessment tool. 

We engaged with hospital stakeholders during the project’s conceptualization phase as well as throughout the project operation. We also actively attended digital health technology workshops to engage with government authorities and policy-makers including the Vietnamese health ministry.

## Relevant changes

We are working on refining the designs of the digital health technologies in our project. Based on patient feedback, we are altering the wearable sensor to increase comfort. After the workshop participants had selected the core functionalities of the assessment tool, we built the user interface, which subsequently was tested for usability in an iterative manner by the clinical staff members.

## Lessons learnt

### Data

Despite the potential of using artificial intelligence models developed from pooled data, we found seasonality and disease prevalence affected our model performance.^8^ These findings highlight the need for continuous data collection methods to sustain model performance for dengue tools. As the data availability and quality issues hindered the application of automated pipelines for data collection, pre-processing and sharing in our project, we emphasize the importance of strengthening the capacity for electronic medical records transition and interoperability. We are therefore facilitating the upgrade of current hospital electronic medical records to comply with the HL7 Fast Healthcare Interoperability Resources, an international industry standard, which will facilitate health data exchange across different systems.

Furthermore, we acknowledged the use of large secondary data sets for model development could introduce hidden biases that are difficult to detect and account for in our models.^9^ While patient diversity and large cohort size from pooled data were beneficial in providing a representative sample over time, the narrow scope and homogenous nature of research data can adversely affect the downstream generalizability and real-world performance of artificial intelligence models.^9^ We are therefore continuing to refine our models using data from our prospective ongoing studies to validate the clinical utility and generalizability, and to reduce biases. Furthermore, population groups underrepresented or excluded in research data represent significant challenges to address health inequity.^10^ Therefore, continuous real-world evaluation is needed to ensure clinical utility and improve the performance through continuous learning.

### Implementation

New digital health technologies need to be sustainable, pragmatic, interoperable and aligned with existing health-care processes,^11^ as well as solving real-life problems that matter to the population. To ensure that the project fulfilled these points, we discussed with stakeholders before the implementation, and conducted several activities throughout the development of the digital health technologies. Interviews with individual health-care workers around decision-making processes during the development revealed insights about the usability of the clinical decision support systems, and the need to balance clinical usefulness and digital complexity of the tool. Clinicians highlighted that digital health technologies should not significantly alter existing pathways in terms of data entry and patient flow.^7^ As process mapping revealed different patient flow between adult and paediatric patients, the implementation of the assessment tool should thus have flexibility according to the age of the patient.^7^ Data entry to two electronic systems (hospital electronic medical records and the assessment tool) would discourage uptake of the new tool in clinical practice. We therefore recommend developers of digital health technologies to consider strategies for system integration into existing electronic medical records to maximize uptake and sustainability. Furthermore, to increase long-term uptake, we asked about patients’ perceptions towards the wearable sensor, and we learnt that the patients and their relatives expressed concerns that its use would reduce interactions with health-care workers. Although digital literacy and the role of technology in many low- and middle-income country settings are increasing at a significant pace, better understanding of attitudes towards digital health technologies is needed. An implementation research approach is therefore crucial in any digital health technology project, and should ideally run alongside development of the technology to better guide the iterative development and delivery.

During the project we had difficulties in recruiting local artificial intelligence researchers, hence local research capacity needs to be boosted through collaboration with local academic institutions, as well as investment in higher education. Another important and complex challenge to the viability of many digital health technology projects in low-resource settings is the migration of digital practitioners to high-income settings.

### Regulation

Recent released policies focusing on enhancing electronic medical records interoperability, picture archiving, and systems to support communications between health-care bodies,^12^ provide an opportunity to develop digital health technologies within our setting. These policies facilitate the digital transformation, by increasing data availability and interoperability between systems. We engaged with government authorities and policy-makers, including the health ministry, early on in our project to better understand the priorities and infrastructure investment. We found that policy-makers were receptive to our experience and supported plans on future roll-out.

Artificial intelligence-enhanced applications in health care can only create impact providing they exist within a legal and regulatory framework. Understanding these environments is important in facilitating collaboration and safeguarding users.^4^ In Viet Nam, we have found that the availability of local guidelines supporting development of novel digital health technologies is limited. Adapting existing guidance on artificial intelligence developed in high-income settings can provide important insights,^13^^,^^14^ although all stakeholders involved need to be cognizant of the relevance and direct applicability of the guidance. Given the fast pace of digital health technology development in Viet Nam, it is important to have a formal, iterative process led at the national level, to capture local needs and develop necessary regulations.^12^ At the same time, heterogeneity between individual health-care settings requires that sufficient support and expertise are available for local bodies (such as research ethics committees) for evaluation of novel digital health technologies.

The project is still ongoing, and the next steps in our dengue work package are to refine the design of the wearable sensor based on feedback from patients; validate the artificial intelligence models embedded in the assessment tool; and further explore potential pathways for regulatory submission.

The lessons learnt (Box 1) from our project, as well as the challenges and potential solutions in applying digital health technologies (Table 1) in Viet Nam could be useful for other settings in low- and middle-income countries. We suggest that challenges specific to low- and middle-income countries need to be explicitly considered, and often require a different approach than replicating or extrapolating the solutions from the high-income countries. Application of artificial intelligence and digital health technologies requires development of digital infrastructure, placing local health-care workers and stakeholders at the centre of the implementation process, and co-development of regulatory frameworks across different relevant bodies.

Box 1 **Summary of main lessons learnt**

- An interoperable and appropriate plan for data management including collection, sharing and integration is important.
- Engagements and implementation studies should be conceptualized and conducted alongside the digital health technology development.
- The priorities of end-users and understanding context and regulatory landscape are crucial for success.

**Table 1:** Challenges and potential solutions in applying digital health technologies, lessons from Viet Nam

Challenges, by stage	Potential solutions
**Data and development**	
• Limited data availability for model development • Ensuring representative data is available, and model fairness	• Strengthening capacity for data collection and ensuring system interoperability • Multidisciplinary collaboration (clinical, data science, information technology) for development work; support local workforce development and retention; and specific focus on addressing model bias
**Implementation**	
• Difficulties to integrate digital health technologies in existing health-care systems • Enhancing uptake and behaviour change • Addressing concerns of end-users and bodies regarding familiarity of using digital health technologies in local settings	• Co-design of interventions with focus on integration and minimizing disruption to workflow. • Better understanding of health-care context through methods including process mapping and human-centred design • Interventions targeting digital literacy and engagement with relevant communities. Involve local institutions and policy-makers during public engagement
**Legal and regulatory framework**	
• Lack of artificial intelligence governance capability and local expertise • Limited experience with intellectual property • Varying digital ecosystem maturity	• Initial adoption of existing guidelines while collaborating with stakeholders to continuously re-evaluate their relevance to local setting • Adoption of flexible frameworks and early engagement in intellectual property to ensure clear understanding of rights between stakeholders • Sharing experiences with regulatory bodies and using research findings to inform and co-develop policies relevant to the setting
